# A hierarchical procedure to select intrauterine and extrauterine factors for methodological validation of preterm birth risk estimation

**DOI:** 10.1186/s12884-021-03654-3

**Published:** 2021-04-16

**Authors:** Pasquale Anthony Della Rosa, Cesare Miglioli, Martina Caglioni, Francesca Tiberio, Kelsey H.H. Mosser, Edoardo Vignotto, Matteo Canini, Cristina Baldoli, Andrea Falini, Massimo Candiani, Paolo Cavoretto

**Affiliations:** 1 Neuroradiology Department, IRCCS San Raffaele Hospital and University, via Olgettina 62, Milan, 20132 Italy; 2 Research Center for Statistics, University of Geneva, Boulevard du Pont-d’Arve 40, Geneva, 1205 Switzerland; 3 Obstetrics and Gynaecology Department, IRCCS San Raffaele Hospital and University, via Olgettina 62, Milan, 20132 Italy

**Keywords:** Preterm delivery, Pregnancy, Risk factors, Intrauterine, Extrauterine, Akaike information criterion, Random forest, Fuzzy clustering, Precision medicine

## Abstract

**Background:**

Etiopathogenesis of preterm birth (PTB) is multifactorial, with a universe of risk factors interplaying between the mother and the environment. It is of utmost importance to identify the most informative factors in order to estimate the degree of PTB risk and trace an individualized profile. The aims of the present study were: 1) to identify all acknowledged risk factors for PTB and to select the most informative ones for defining an accurate model of risk prediction; 2) to verify predictive accuracy of the model and 3) to identify group profiles according to the degree of PTB risk based on the most informative factors.

**Methods:**

The Maternal Frailty Inventory (MaFra) was created based on a systematic review of the literature including 174 identified intrauterine (IU) and extrauterine (EU) factors. A sample of 111 pregnant women previously categorized in low or high risk for PTB below 37 weeks, according to ACOG guidelines, underwent the MaFra Inventory. First, univariate logistic regression enabled *p*-value ordering and the Akaike Information Criterion (AIC) selected the model including the most informative MaFra factors. Second, random forest classifier verified the overall predictive accuracy of the model. Third, fuzzy c-means clustering assigned group membership based on the most informative MaFra factors.

**Results:**

The most informative and parsimonious model selected through AIC included Placenta Previa, Pregnancy Induced Hypertension, Antibiotics, Cervix Length, Physical Exercise, Fetal Growth, Maternal Anxiety, Preeclampsia, Antihypertensives. The random forest classifier including only the most informative IU and EU factors achieved an overall accuracy of 81.08% and an AUC of 0.8122. The cluster analysis identified three groups of typical pregnant women, profiled on the basis of the most informative IU and EU risk factors from a lower to a higher degree of PTB risk, which paralleled time of birth delivery.

**Conclusions:**

This study establishes a generalized methodology for building-up an evidence-based holistic risk assessment for PTB to be used in clinical practice. Relevant and essential factors were selected and were able to provide an accurate estimation of degree of PTB risk based on the most informative constellation of IU and EU factors.

**Supplementary Information:**

The online version contains supplementary material available at (10.1186/s12884-021-03654-3).

## Background

### Etiologic vs generalized prediction of PTB

Preterm Birth (PTB) affects 5 to 18% of pregnancies and it is the leading cause of neonatal death [1]. The major etiological dichotomy of PTB involves primarily iatrogenic (iPTB) or spontaneous (sPTB) components, arising generally from different pathophysiology. However, differentiation is often difficult and clear phenotypic and etiologic classification of PTB remains a controversial issue [2]. Etiopathogenesis of PTB is multifactorial and factors possibly contributing to but not completely explaining PTB include: obstetrical and gynecological history (i.e. prior PTBs, short cervical length), chronic medical conditions (i.e. arterial hypertension, diabetes mellitus) and pregnancy complications (i.e. preeclampsia, urinary tracts infections) [3]. A number of other risk factors for PTB have been globally identified and their relevance is broadly acknowledged including: a history of pregnancy complications [4], placental abnormalities or abruption [5], preeclampsia [6], cigarette smoking [7], infections [8] and fetal growth restriction [9–11]. However, little is known about the interplay of these components along with other environmental and social factors. Previous studies produced solid evidence for Bayesian PTB risk estimation based upon history, specific clinical features or biomarkers, for both iPTB or sPTB [12–14]). However, these prediction models, despite remarkable performances are limited to the capacity of prediction within the mechanisms for which they were defined and tested. Given the etiologic diversity of PTB it has been referred to as the “great obstetrical syndrome” or “Preterm Birth Syndrome” [15], characterized by both uterine and extrauterine components. There is an urgent need of holistic-generalized prediction models of PTB, capable of encompassing all or most etiologic mechanisms of PTB, in order to provide a useful tool at the bedside for clinical decision purposes. Therefore, we describe here the method of definition of a multi-layer prediction model, suitable for assisting clinicians in planning patients monitoring or treatment(s). Quantification and assessment of risk factors is a clinically established method to characterize the risk of PTB as defined by ACOG [16, 17]. However, it is currently difficult to translate this information into a comprehensive risk assessment capable of attributing to different factors or clusters specific weights, thus guiding a patient-specific approach. This risk individualization would lead to definite managements and treatments within a precision medicine approach.

### Study aims and objectives

The primary aim of this study was to identify all available risk factors for PTB and to select the most relevant among the overall group for the scope of defining accurate risk prediction. This primary aim was pursued first by quantifying the risk of PTB associated with intrauterine (IU) and extrauterine (EU) factors separately to evaluate the contribution of factors in each gestational dimension, and then by searching for the most informative risk factors and select the best model including both IU and EU factors with the greatest contribution for the degree of estimated PTB risk. The second purpose of this study was to verify the predictive accuracy of the model including solely the most informative IU and EU risk factors of PTB birth identified at the previous step, as compared to a model including all the IU and EU risk factors considered in the present study in order to pinpoint the PTB constellation of risk factors according to which clinicians may attribute the degree of estimated PTB risk and increase confidence in managing potential PTB. The ultimate objective of this study was to overcome the high/low risk dichotomy of PTB risk through a clustering approach in order to verify if our sample of pregnant women may distribute in more than 2 groups based on the most informative IU and EU risk factors. Such an approach may allow us to assign to each pregnant woman a group membership probability value based on relevant risk factors able to define potential group boundaries along a continuum that moves from a lower to a higher degree of PTB risk. Such a gestational constellation of both IU and EU factors defining PTB risk, may set the basis for future creation of a clinical assessment tool reliably evaluating and quantifying the patient-specific risk for PTB that could indicate in each specific clinical case the most effective clinical management including: monitoring, surveillance, prophylaxis or therapy.

## Materials and methods

### Participants

This study was based on data collected from pregnant women (n = 116) enrolled in the Obstetrics Gynecology Department of the San Raffaele Hospital in Milan, between May 2019 and December 2019. The patients who met the inclusion criteria were informed on the aim of the study, the Maternal Frailty Inventory (see below for details) administration and the time commitment necessary and were asked if they wanted to participate. The criteria for inclusion were: mothers with a single fetus, gestational age below 37 weeks. The exclusion criteria were: fetuses with prenatal diagnosis of major malformation with subsequent medical abortion, twin pregnancies, pregnant women who did not sign the informed consent. All pregnant women gave written informed consent before inclusion in the study and research was performed in compliance with the principles of the Helsinki Declaration, and was approved by the Ethics Committee of the San Raffaele Hospital, Milan. STROBE guidelines for cross-sectional studies were followed [18]. A final sample of 111 pregnant women, ranging in age from 16 to 45 years with a mean age of 32.5 years (standard deviation 6.05) was included in the present study.

### Risk factors definitions

**Fig. 1:**
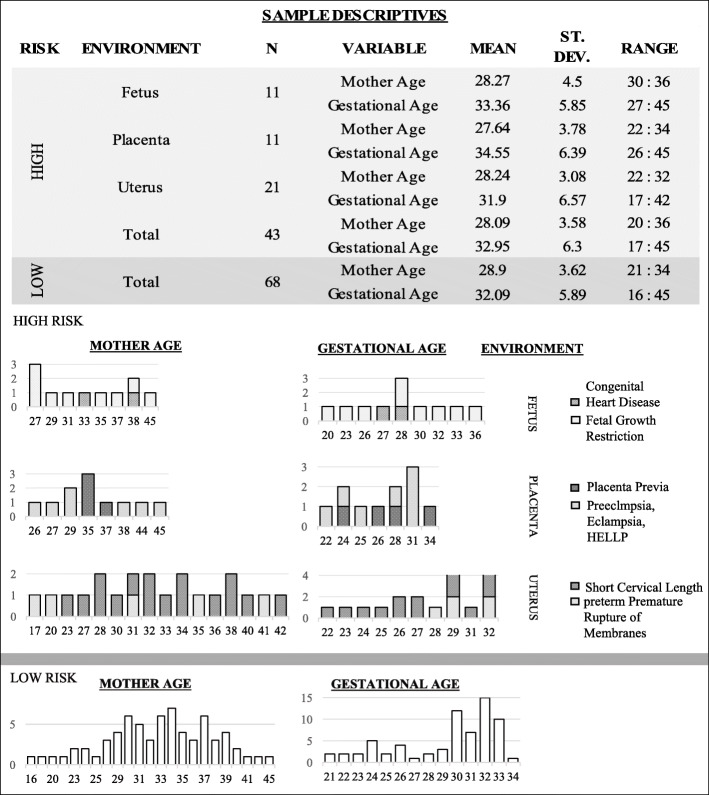
Risk factors distribution. We show the distribution of risk factors in the sample of pregnant women according to maternal age and fetal gestational age

We classified the sample of 111 pregnant women in low (i.e. n=68) or high risk (i.e. n=43) for PTB, according to the 2012 ACOG clinical management guidelines [17], considering risk factors related to obstetrical and gynecological history and pregnancy complications. Each risk factor has been labeled in relation to the environment from which it originates: uterus (U), placenta (P), fetus (F), or extrauterine indicating those clinical conditions that do not originate from the utero-placenta-fetal system. Both mother age (p=.075) and gestational age (p=.71) did not significantly differ between low and high risk subsamples of pregnant women (see Table 1 and Fig. 1). PTB was defined as any delivery below 37 weeks and 0 days of gestational age, regardless of the etiology (spontaneous or iatrogenic). Dating of pregnancy was confirmed in all cases included in the study with ultrasound crown-rump length measurements obtained in the first trimester. Fetal growth restriction was defined in presence of fetal biometry below the 3rd centile for gestational age or, with a drop in abdominal circumference below the 10th centile or EFW of >2 quartiles or >50 percentiles and/or abnormalities of fetal or uterine Doppler [11]. Pregnancy induced hypertension was defined as systolic blood pressure ≥140 mmHg and/or diastolic blood pressure ≥90 mmHg developing after 20 weeks of gestation, whereas hypertension that antecedes pregnancy or was present on at least two occasions before the 20th week of gestation or persists longer than 12 weeks postpartum was defined as chronic hypertension. Preeclampsia was defined as a new onset of hypertension and proteinuria or a new onset of hypertension and significant end-organ dysfunction with or without proteinuria after 20 weeks of gestation or postpartum in a previously normotensive woman [23]. Short cervix was defined in presence of transvaginal ultrasound measurement cervical length below 15 mm at 20-24 weeks [28]. Polydramnios was defined as a deepest amniotic fluid above 8 cm, oligohydramnios as an amniotic fluid index below 5 cm. Placenta previa was defined when the lower edge of the placental insertion was distant from the internal os ≤15 mm at time of delivery, and abruption placentae as any hemorrhage above 20 weeks trimester resulting in the evidence of bleeding from the placental bed.

**Table 1:** Risk Factors preterm birth (PTB). Risk factors for PTB used for sample classification in low/high PTB risk and their definitions

Risk factors	Definition	Uterine environment	Study
Short Cervical Length	Transvaginal ultrasound cervical length ≤ 25 mm (2nd to 3rd centile)	U	[19]
Polyhydramnios Severe	Single deepest pocket (SDP) ≥ 16.0 cm or amniotic fluid index (AFI) > 35.0 cm	U	[20]
pPROM	Preterm prelabor rupture of membranes	U	[21]
Medically Assisted Procreation	All the methods or techniques based on the manipulation of reproductive cells (gametes) that will allow infertile couples to conceive a child	U	[22]
Prior PTB	Previous delivery that occurs between 20 and 37 weeks of gestation	U	[4]
Pregnancy Induced Hypertention (PIH)	Systolic blood pressure ≥ 140 mmHg or diastolic blood pressure ≥ 90 mmHg on at least 2 occasions at least 4 hours apart after 20 weeks of gestation in a previously normotensive patient	P	[23]
Placenta Previa	Placenta that completely or partially covered the internal os on a second- or third-trimester imaging study	P	[24]
Placental Abruption	Partial or complete placental detachment prior to delivery of the fetus	P	[5]
Preeclampsia, Eclampsia, HELLP	New onset of hypertension and proteinuria or hypertension and end-organ dysfunction with or without proteinuria after 20 weeks of gestation in a previously normotensive woman	P	[23]
Fetal Growth Restriction	EFW < 3 centile or EFW < 10 centile with Doppler abnormalities on maternal or fetal side or decline in EFW	F	[25]
Urinary Tract Infections (UTI)	Cystitis (infection of the bladder/lower urinary tract) and pyelonephritis (infection of the kidney/upper urinary tract) n pregnant women	E	[26]
Complex Autoimmune Diseases With Polytherapy	i.e. Systemic lupus erythematosus (LES), Antiphospholipid Syndrome (APS)	E	[27]

### Maternal frailty inventory of risk factors (MaFra)

In order to identify a maternal frailty for PTB, we developed the Maternal Frailty (MaFra) Inventory, identifying risk factors as IU or EU [29]. IU factors encompass all aspects of the uterine environment (i.e. exclusively uterus and its contents including the utero-placenta-fetal system) whereas EU factors involve all other aspects arising from extrinsic factors or different anatomical regions (e.g. systemic infections, autoimmune syndromes, medi- cations, stress or anxiety, etc). A systematic search of PubMed, CINAHL, Google Scholar and PsycINFO was performed in accordance with a detailed search strategy in order to pinpoint all relevant IU and EU PTB risk factors. All published citations were cross-referenced from other relevant studies. Studies that investigated the effects or the association between maternal gynecological, obstetrical, environment, mental state, lifestyle and any other socio-demographical factor associated with PTB were eligible for inclusion. Two authors (MC and FT) independently reviewed all studies to identify eligible studies for each risk factor included in the MaFra inventory. We included studies published in English only, from January 1990 through September 2019. We included systematic reviews, meta-analysis, randomized controlled trials, cohort, case-control and cross-sectional studies in which PTB is the outcome of interest. The reference lists of review articles and relevant meta-analyses were checked for additional references. Only peer-reviewed literature was included. Studies from high-income, middle-income and developing countries were all included. In addition, reported epidemiological data by national and international organizations [30–33] was accounted for risk factor inclusion. Finally, The MaFra Inventory comprises 174 variables encompassing all intrauterine and extra-uterine factors included and referenced in (Table 2).

**Table 2:** Maternal Frailty (MaFra) Inventory. Intrauterine and extrauterine preterm birth risk factors included in the MaFra Inventory. 150 items assess the 71 listed factors while 24 Items collect more general sociodemographic, anamnestic and basic pregnancy history information for a total of 174 items included in the MaFra Inventory

System	Pregnancy anamnesis	Factors	Number of items	High/ low risk	Uterine environment	Study
UTERINE	PREVIOUS (Conditions of Pregnancy)	Parity	1	L	U	[34]
UTERINE	PREVIOUS (Conditions of Pregnancy)	Delivery Onset	2	L	U	[35]
UTERINE	PREVIOUS (Conditions of Pregnancy)	Breastfeeding	1	L	U	[36]
UTERINE	PREVIOUS (Conditions of Pregnancy)	Multiple Gestation	1	L	U	[37]
UTERINE	PREVIOUS (Conditions of Pregnancy)	Voluntary Interruption of Pregnancy	1	L	U	[38]
UTERINE	PREVIOUS (Conditions of Pregnancy)	Miscarriage	1	L	U	[38]
UTERINE	PREVIOUS (Conditions of Pregnancy)	Prior PTB	2	H	U	[4]
UTERINE	CURRENT (Conditions of Pregnancy)	Fetal Growth Restriction	9	H	F	[9]
UTERINE	CURRENT (Conditions of Pregnancy)	Medically Assisted Procreation	2	H	U	[22]
UTERINE	CURRENT (Conditions of Pregnancy)	Short Cervical Length	1	H	U	[19]
UTERINE	CURRENT (Conditions of Pregnancy)	Placenta Previa	1	H	P	[24]
UTERINE	CURRENT (Conditions of Pregnancy)	Placental Abruption	1	H	P	[5]
UTERINE	CURRENT (Conditions of Pregnancy)	Uterine Fibroid (Leiomyomas)	1	L	U	[39]
UTERINE	CURRENT (Conditions of Pregnancy)	Polidramnios	1	H	U	[20]
UTERINE	CURRENT (Conditions of Pregnancy)	Oligoidramnios	1	L	U	[40]
UTERINE	CURRENT (Conditions of Pregnancy)	Fetal Fibronectin	1	H	F	[41]
UTERINE	CURRENT (Conditions of Pregnancy)	Interleukin (IL)-6-Inflammatory Cytokine	1	H	U	[42]
UTERINE	CURRENT (Conditions of Pregnancy)	Fetal Sex	1	L	F	[43]
UTERINE	CURRENT (Conditions of Pregnancy)	Pregnancy Induced Hypertension (PIH)	1	H	P	[44]
UTERINE	CURRENT (Conditions of Pregnancy)	Preeclampsia	3	H	P	[45]
EXTRAUTERINE	CURRENT (Conditions of Pregnancy)	Pregnancy Awareness	10	L	-	[46]
EXTRAUTERINE	CURRENT (Conditions of Pregnancy)	Obesity	4	L	-	[47]
EXTRAUTERINE	CURRENT (Conditions of Pregnancy)	Gestational Diabetes	2	L	-	[48]
EXTRAUTERINE	CURRENT (Conditions of Pregnancy)	Thyroid Disease	2	L	-	[49]
EXTRAUTERINE	CURRENT (Conditions of Pregnancy)	Autoimmune Syndrome	1	H	-	[27]
EXTRAUTERINE	CURRENT (Conditions of Pregnancy)	Urinary Tract Infection (UTI)	1	H	-	[26]
EXTRAUTERINE	CURRENT (Conditions of Pregnancy)	Fever	1	L	-	[50]
EXTRAUTERINE	CURRENT (Conditions of Pregnancy)	Rubella	1	L	-	[51]
EXTRAUTERINE	CURRENT (Conditions of Pregnancy)	Infection	4	L	-	[52]

**Table 2:** Maternal Frailty (MaFra) Inventory. Intrauterine and extrauterine preterm birth risk factors included in the MaFra Inventory. 150 items assess the 71 listed factors while 24 Items collect more general sociodemographic, anamnestic and basic pregnancy history information for a total of 174 items included in the MaFra Inventory (*Continued*)

System	Pregnancy anamnesis	Factors	Number of items	High/ low risk	Uterine environment	Study
EXTRAUTERINE	CURRENT (Conditions of Pregnancy)	Corticosteroids	4	L	-	[53]
EXTRAUTERINE	CURRENT (Conditions of Pregnancy)	Analgesics	1	L	-	[54]
EXTRAUTERINE	CURRENT (Conditions of Pregnancy)	Antihypertensives	1	L	-	[55]
EXTRAUTERINE	CURRENT (Conditions of Pregnancy)	Antiemetics	1	L	-	[56]
EXTRAUTERINE	CURRENT (Conditions of Pregnancy)	Antihistamines	1	L	-	[57]
EXTRAUTERINE	CURRENT (Conditions of Pregnancy)	Anti-inflammatories	1	L	-	[58]
EXTRAUTERINE	CURRENT (Conditions of Pregnancy)	Hormones	1	L	-	[59]
EXTRAUTERINE	CURRENT (Conditions of Pregnancy)	Vaccinations	1	L	-	[60]
EXTRAUTERINE	CURRENT (Conditions of Pregnancy)	Antibiotics	1	L	-	[61]
EXTRAUTERINE	BEFORE (Lifestyle)	Folic Acid Supplementation	3	L	-	[62]
EXTRAUTERINE	BEFORE (Lifestyle)	Estroprogestinic Therapy	1	L	-	[63]
EXTRAUTERINE	BEFORE (Lifestyle)	Diabetes	1	L	-	[64]
EXTRAUTERINE	BEFORE (Lifestyle)	Hypertension	1	L	-	[64]
EXTRAUTERINE	BEFORE (Lifestyle)	Maternal Medication	2	L	-	[65]
EXTRAUTERINE	BEFORE (Lifestyle)	Cigarette Smoking	2	L	-	[7]
EXTRAUTERINE	BEFORE (Lifestyle)	Alcohol use	2	L	-	[66]
EXTRAUTERINE	BEFORE (Lifestyle)	Use of Drugs/Substance Abuse	6	L	-	[67]
EXTRAUTERINE	BEFORE (Lifestyle)	Caffeine	2	L	-	[68]
EXTRAUTERINE	BEFORE (Lifestyle)	Maternal Stress	1	L	-	[69]
EXTRAUTERINE	BEFORE (Lifestyle)	Weight	1	L	-	[70]

### Data preprocessing

After the data collection process, data cleaning procedures implied that all the variables with a) more than 50% of missing values or b) less than 3 positive occurrences for any dichotomic categorical variable (e.g. no subject has declared use of bronchodilators or HIV infection) were not taken in consideration for further analyses steps with more than half subjects with missing values. Along the same lines, we collapsed nested categorical variables (i.e. that depend on a previous positive answer to another question) and aggregated levels whenever a) or b) applied. After data cleaning, a subset of 86 variables of the original 174 were retained for all analyses steps. The median non-response rate for each variable, considering also missing values, was 0.9% while the average is 6%. To constitute the final dataset, we use random forest imputation (see e.g. [89] for an overview) to avoid slicing effects (i.e. NAs in different places for different variables that inevitably reduce the sample size) at the modeling step of the explanatory phase. This procedure is commonly considered robust especially in a low/moderate non response framework as the one of our study.

## Data analyses

### Explanatory phase

We performed a univariate logistic regression (see ch. 6 in [90] for a detailed overview) for each variable and ordered the risk factors based upon the associated *p*-values, from the smallest to the biggest. To avoid the potential imputation bias on the ordering procedure, we make use of the unimputed Mafra dataset. In order to deal with perfect separation (see e.g. [91]) (i.e. all subjects showing this risk factor labelled as high PTB risk) we used a bias reduction technique [92] leading to increased accuracy of all the estimates. To select the model including the most informative MaFra factors, we use AIC (i.e. Akaike Information Criterion see [93]) which is a well-known model selection criterion (see e.g. [94] ch. 2 and 3 for a detailed discussion). Based on the *p*-value ordering, relevant factors were added in a model one at a time and for each successive logistic regression model an AIC value was calculated. The model search ended when the sequence of AIC values reached its minimum, since further addition of other risk factors to the model increased the AIC. Model selection was performed including a) IU factors only b) EU factors only and c) including both IU and EU factors. At this modeling stage, to avoid slicing effects, we use the imputed dataset as described in the preprocessing section. To summarize, the aim of the explanatory phase was to select the most informative risk factors, representative of the Mafra inventory. Thus, *p*-value ordering was coupled with a model selection criterion to stop the search in order to select a group of variables which best explain PTB risk in our sample. Finally, we analyzed two models, based on the distinction between IU and EU risk factors, to understand the specific contribution of the two gestational dimensions and a third model which combined both IU and EU risk factors to identify the most informative factors irrespective of gestational dimension. For this combined evaluation, AIC differences were also used to assess the level of empirical support of a given model with respect to the best model (i.e. the one that reaches minimum AIC). The i-th AIC difference, between the i-th model and the best model, was calculated as *Δ*_*i*_=*AIC*_*i*_−*AIC*_*min*_. Starting from values of *Δ*_*i*_>4, the level of empirical support for the i-th model, with respect to the best model is considerably less (see [94] ch. 2 for a detailed explanation).

### Predictive phase

We tested the ability of the most informative IU and EU factors selected in the explanatory phase to summarize the MaFra Dataset in a classification task. In particular we compared the out of sample classification error of a random forest [95] trained with all the MaFra risk factors against a random forest trained only with the subset of risk factors determined by AIC. We estimated the classification error by means of leave-one-out-cross-validation (i.e. LOOCV see ch. 7 in [96] for a detailed explanation). In our case, it consisted of leaving one mother out as a *test set* and predicting her degree of PTB risk with random forest trained on the other 110 remaining mothers. We did this for every subject to obtain the desired estimate of the out of sample classification error for both competitors. The aim of this predictive phase was to understand if the subset of IU and EU factors, selected by AIC in the explanatory phase, reached a comparable or higher overall classification accuracy than the entire pool of both IU and EU risk factors included in the MaFra Inventory.

### Clustering phase

We evaluated the degree of PTB risk with fuzzy C-means (i.e. FCM [97]) clustering of the most informative IU and EU factors selected in the explanatory phase and used for classification in the predictive phase. The main steps in the FCM algorithm included: (1) fix a number of clusters and assign coefficients randomly to each data point for being in one of the clusters; (2) compute the centroids (i.e. mean of all points, weighted by their degree of being into the cluster) and, for each data point, update the coefficients; (3) repetition until convergence.

## Results

### Explanatory phase

**Fig. 2:**
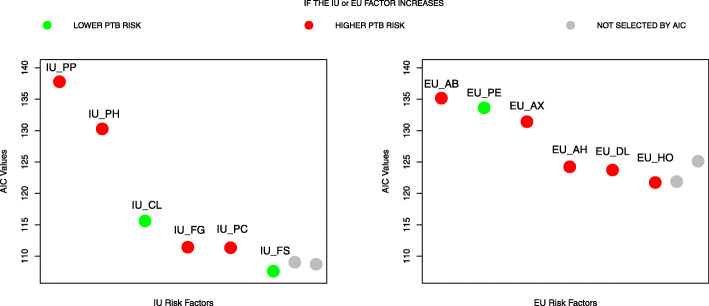
Akaike Information Criterion (AIC) Model Selection for intrauterine (IU) and extrauterine (EU) risk factors. Forward selection of both IU and EU most representative risk factors by AIC. On the x-axis we display the labels of the risk factors entered with respect to the order of Table 3; on the y-axis the AIC values. Each point on the graph represents the AIC value scored by a model composed by all the risk factors on the left of the point (including the label above the point). The green color represents a decrease in the expected preterm birth risk whenever the specific risk factor augments holding the other variables constant at a certain value (e.g. increasing the cervical length IU_CL leads to a lower expected risk of preterm birth). For dichotomous variables, the variation is from absence to presence (e.g. for EU_PE, doing physical exercise decrease the expected preterm birth risk). In the specific case of IU_FS (Fetal Sex), the variation is from male to female. On the other hand, the red color represents an increase in the expected preterm birth risk whenever a specific dichotomous risk factor becomes active holding the other variables constant at a certain value (e.g. for EU_AB, giving antibiotics leads to an expected increase of the preterm birth risk). Both colors directly reflect the signs of the beta coefficients associated to each risk factor in the specific logistic regression model. Finally the grey colored points represent other possible risk factors that were not added because the criterion had already reached its minimum

**Fig. 3:**
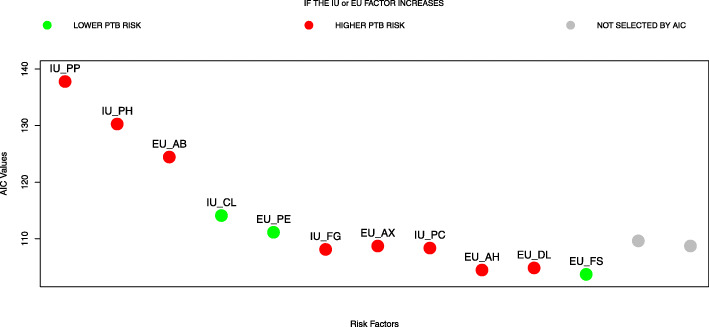
Akaike Information Criterion (AIC) Model Selection for all Maternal Frailty (MaFra) risk factors. Forward selection of the most representative risk factors by AIC in the MaFra dataset. On the x-axis we display the labels of the risk factors entered with respect to the order of Table 3; on the y-axis the AIC values. Each point on the graph represents the AIC value scored by a model composed by all the risk factors on the left of the point (including the label above the point). The green color represents a decrease in the expected preterm birth risk whenever the specific risk factor augments holding the other variables constant at a certain value (e.g. increasing the cervical length IU_CL leads to a lower expected risk of preterm birth). For dichotomous variables, the variation is from absence to presence (e.g. for EU_PE, doing physical exercise decrease the preterm birth risk). In the specific case of IU_FS (Fetal Sex), the variation is from male to female. On the other hand, the red color represents an increase in the expected preterm birth risk whenever a specific dichotomous risk factor becomes active holding the other variables constant at a certain value (e.g. for EU_AB, giving antibiotics leads to an increase of the expected preterm birth risk). Both colors directly reflect the signs of the beta coefficients associated to each risk factor in the specific logistic regression model. Finally the grey colored points represent other possible risk factors that were not added because the criterion had already reached its minimum

The ordered univariate *p*-values of the selected MaFra factors are reported in Table 3. For the model including only IU variables, AIC reached global minimum (i.e. 107.62) with the inclusion of Placenta Previa (PP), Pregnancy Induced Hypertension (PH), Cervix Length (CL), Fetal Growth Restriction (FG), Preeclampsia (PC) and Fetal Sex (FS) (see Fig. 2); for the model including only EU variables, AIC reached global minimum (i.e. 121.74) with the inclusion of Antibiotics (AB), Physical Exercise (PE), Maternal Anxiety (AX), Antihypertensives (AH), Depression Level (DL) and Hormones Medication (HO) (see Fig. 2); for the model including both IU and EU variables, AIC reached a local minimum (i.e. 104.47) including 9 risk factors: PP, PH, AB, CL, PE, FG, AX, PC, AH. On the other hand, a global minimum (i.e. 103.71) is reached with the addition of two more factors, namely DL and FS (see Fig. 3). We compared the two models in terms of AIC differences (*Δ*_*i*_=*AIC*_*i*_−*AIC*_*min*_ see [94] ch.2 for a detailed explanation). There was substantial empirical support (i.e. *Δ*_*i*_<4) for the models with respectively 9 and 11 risk factors. There was also evidence (see Table 4) that adding the risk factors DL and FS does not bring additional explanatory power since the models are almost equivalent in terms of AIC. Thus we focused on the model with 9 risk factors, 5 IU and 4 EU, which is the most parsimonious. Since the role EU factors in explaining and predicting PTB risk is not clear-cut through the literature and thus requires validation and replicability from further studies, we also present a detailed overview of the 4 EU factors selected by AIC in the Supplementary material.

**Table 3:** Ordered univariate logistic regression *p*-values of the risk factors below the 0.05 threshold

Risk Factors	Risk Factor Number	Label	*P*-value
Placenta Praevia	1	IU_PP	0
Pregnancy Induced Hypertension	2	IU_PH	0
Antibiotics Medication	3	EU_AB	0.00041
Cervical Length	4	IU_CL	0.00247
Physical Exercise	5	EU_PE	0.00822
Fetal Growth Restriction	6	IU_FG	0.00968
Anxiety	7	EU_AX	0.01475
Preeclampsia	8	IU_PC	0.01840
Antihypertensive Medication	9	EU_AH	0.02568
Depression Level	10	EU_DL	0.04306
Fetal Sex	11	IU_FS	0.04581
Hormones Medication	12	EU_HO	0.06358

The last risk factor, hormones medication, is the first one above the threshold

**Table 4:** Akaike information criterion (AIC) differences among the candidate nested models presented in Fig. 3

Added Risk Factor	Number of Risk Factors	AIC Value	*Δ*_*i*_
Preeclampsia	8	108.36	4.65
Antihypertensive Medication	9	104.47	**0.76**
Depression Level	10	104.84	**1.13**
Fetal Sex	11	103.71	**0**
Hormones Medication	12	109.62	5.91

We focus on models from size 8 to 12 risk factors. The AIC difference is calculated as *Δ*_*i*_=*AIC*_*i*_−*AIC*_*min*_. Starting from values of *Δ*_*i*_>4 the level of empirical support of model *i*, with respect to the best model, is considerably less (see [94] ch.2 for a detailed explanation). We highlight in bold the AIC differences related to the models for which there is a substantial empirical support (i.e. *Δ*_*i*_<4)

### Predictive phase

**Fig. 4:**
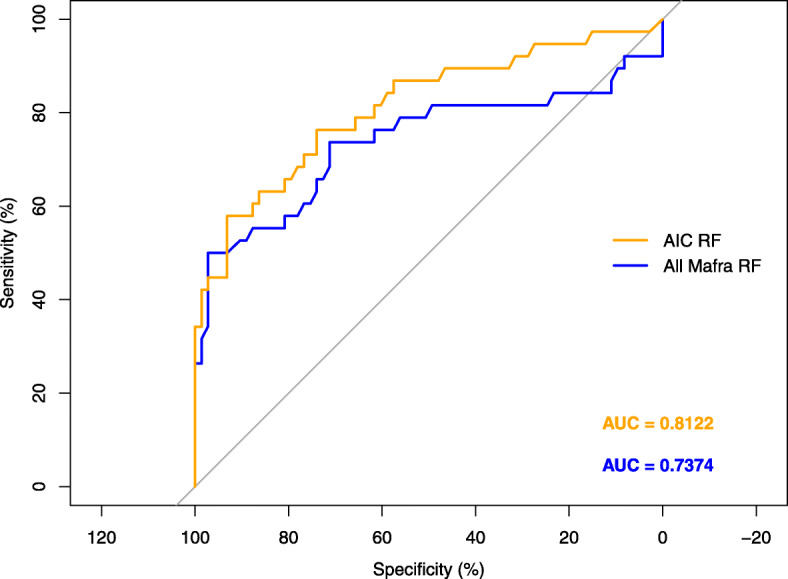
ROC curve comparison between Akaike Information Criterion (AIC) selected and all Maternal Frailty (MaFra) risk factors. We compare the ROC curves of a random forest [95] trained only on the subset of 9 risk factors determined by AIC in the explanatory phase against a random forest trained on all the MaFra risk factors. We estimate the classification error for each competitor by means of leave-one-out-cross-validation (i.e. LOOCV see ch. 7 in [96] for a detailed explanation). We present also the *area under the curve* specific to each competitor in different colors (i.e. orange for the 9 risk factors selected by AIC and blue for the whole set of MaFra risk factors)

**Fig. 5:**
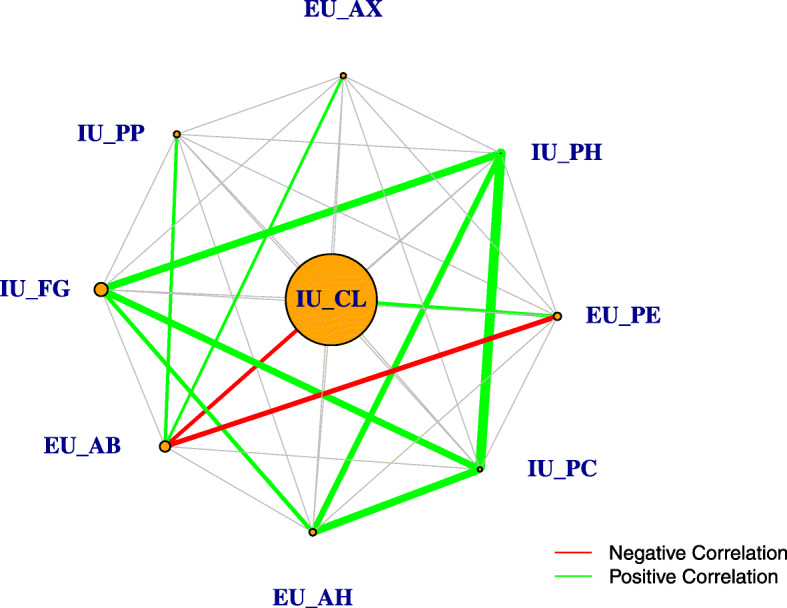
Variable importance network for the risk factors selected by Akaike Information Criterion (AIC). We build a network from the variable importance ([96] ch.15) of a random forest trained on the subset of 9 risk factors determined by AIC in the explanatory phase. The size of each vertex is proportional to the average variable importance while each edge represents the Pearson correlation coefficient between the two risk factors. In addition, the thickness of each edge is proportional to the strength of the correlation and colored edges imply a significant (i.e. *p*_*value*_<0.05) relationship either negative (red edge) or positive (green edge)

In the LOOCV analysis including only the constellation of IU and EU variables identified in the explanatory phase, the random forest classifier achieved a best accuracy of 81.08% and an area under the curve (AUC) of 0.8122. On the other hand, for the LOOCV analysis including the full MaFra Inventory, the random forest classifier yielded a best accuracy of 80.18% and an AUC of 0.7374. We show in Fig. 4 the ROC curves of both classifiers. LOOCV variable importance is summarized in a variable importance network. The size of the vertices are relative to their average permutation score in Random Forest and the edges are related to the correlation structure of these variables (see Fig. 5). In particular, correlation among variables were computed with the point biserial (between continuous and categorical variables) and phi correlation coefficients (between categorical variables) which, in our particular case, are both equivalent to the Pearson correlation coefficient. In addition, correlation coefficients were considered significant at a *p*<0.05 threshold and colored accordingly (see Fig. 5).

### Clustering phase

**Fig. 6:**
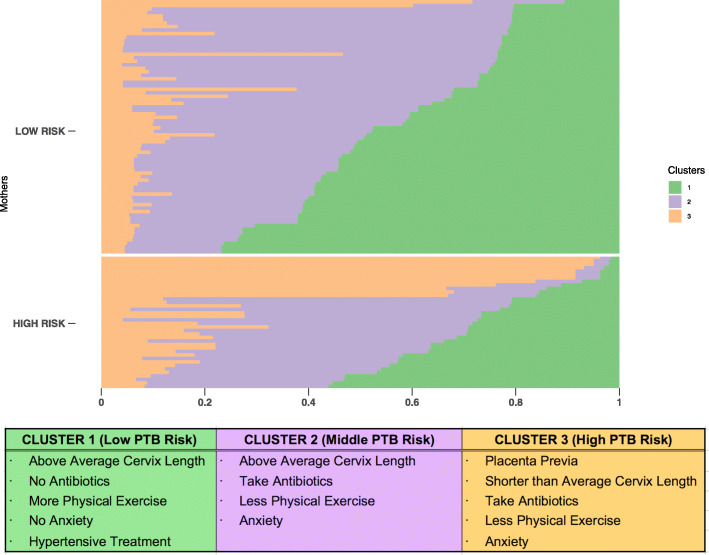
Fuzzy C-means clustering for the risk factors selected by Akaike Information Criterion (AIC) and cluster centroids description. We present three clusters obtained by fuzzy C-means method on the 9 most informative variables selected by AIC at the explanatory phase. We split mothers based on the high/low preterm birth risk classification and we show them in ascending order with respect to cluster 1 membership score (i.e. 0 = no membership; 1 = total membership). We also show the informative preterm birth risk factors for Cluster 1, 2 and 3. The centroids matrix is also provided as a Supplementary material

The selected subset of relevant IU or EU factors that model PTB risk identified three clusters of pregnant women (see Fig. 6). In Cluster 1 (green), the typical woman has a longer than average CL (3.36 cm; average CL: 3.01 cm), has not taken AB, exercises more, is not anxious and has been treated with AH. We refer to this group of women as the low PTB risk cluster. In Cluster 2 (purple) instead the typical woman has taken AB, exercises less and is more anxious than low PTB risk women, however with a longer than average CL (3.24 cm), thus we refer to them as the middle PTB risk cluster. In Cluster 3 (orange) the typical woman presents a shorter than average CL (1.37 cm) than both low and middle risk mothers and PP. Moreover, these women take more AB, are more anxious and exercise less than low PTB risk women. Therefore we refer to them as the high PTB risk cluster. The matrix of centroids derived from fuzzy C-means clustering on the risk factors selected by AIC in the explanatory phase (see Supplementary material) was used to identify means or proportions in each cluster and spot the informative factors which enable grouping interpretation. Furthermore, in order to validate the clustering procedure, we focused on a subset of hard clustered subjects (Cluster 1: *n*=6; Cluster 2: *n*=8; Cluster 3: *n*=9) with coefficients of cluster membership above 0.7, showing the characteristics outlined above for each PTB degree of risk cluster. Namely, we assessed the time of delivery at birth for these women in order to parallel PTB risk cluster membership with birth gestational week. Time delivery of birth differed on average for Clusters 1, 2 and 3 (see Table 5). Namely, gestational week at birth decreases on average with degree of PTB risk cluster membership (i.e. Cluster 3 (High PTB Risk) < Cluster 2 (Middle PTB risk) < Cluster 1 (Low PTB risk) (see Table 5 for all cluster descriptive values). Preterm birth (<37 weeks of gestation) for hard clustered subjects included in the cluster validation procedure was for the larger part spontaneous (sPTB) (n=7) rather than elective (iPTB) (n=2).

**Table 5:** Summary statistics of the gestational week (GW) related to a subset (Cluster 1: *n*=6; Cluster 2: *n*=8; Cluster 3: *n*=9) of hard clustered subjects (i.e. with membership score greater or equal than 0.7)

Cluster	Min GW	1st Qu. GW	Median GW	Mean GW	Sd GW	3rd Qu. GW	Max GW
1	38.00	40.00	40.00	39.93	1.03	40.45	41.00
2	32.20	37.75	38.65	37.68	2.31	38.92	39.00
3	29.00	35.00	36.40	36.31	3.79	38.60	40.70

We obtained the three clusters by fuzzy C-means on the risk factors selected by AIC

## Discussion

### Explanatory phase

First of all, this study pinpoints relevant clinical risk factors related to prematurity within a comprehensive list of available factors described in the Maternal Frailty inventory classified as IU or EU factors.

#### Intrauterine factors

Within the intrauterine constellation of risk factors, our model found that the elements with the highest probability to predict high-risk of PTB to be: placenta previa (PP), pregnancy induced hypertension (PH), cervix length (CL), fetal growth restriction (FG), preeclampsia (PC) and fetal sex (FS). In particular, if PC, PH and/or FG arise during pregnancy, there is an increased likelihood of PTB since all of these clinical conditions share the same placental etiopathogenetic substrate representing a continuum, inherently characterizing risks for PTB [98, 99]. A connection between FG and PTB has been demonstrated via two case-controlled studies, one conducted in Europe [100] and the other in the USA [101]. In the EUROPOP study, 23% of preterm infants were found to be below the 10th percentile of fetal growth standards [100]. Whereas, Bukowski et al. [101] found that 25% of the fetuses delivered at or before 34 weeks did not reach the 5th percentile, 33% did not reach the 10th percentile, and 60% reached the 25th percentile of their individual growth potential. The association between PTB and FG is well established and recent literature confirms this concept [102, 103]. Independent studies described cervical shortening as a strong risk factor for sPTB both in asymptomatic [28, 104–107] and symptomatic pregnant woman [108]. Moreover, a clear continuous inverse relationship was identified between CL at mid trimester ultrasound assessment and the probability of sPTB, with different cut-offs points proposed as a threshold (from 15 and 25 mm). Some authors spotlight a strong correlation of the association PP-short cervix with PTB risk [24]. Stafford et al. [109] reported that women with PP and CL of 30 mm or less were more likely to deliver before 37 weeks than those with a longer cervix, 69% compared with 21% respectively. Male sex is a risk factor for PTB [43], with noted increased association with hormonal differences between the sexes, relatively higher birth weight, and infection-related pathways [110, 111]. A possible explanation may be the higher presence of inflammatory markers found in male placentas compared with female placentas [112, 113], possibly acting synergistically with placental corticotropin-releasing hormone (CRH) production leading to the initiation of labor.

#### Extrauterine factors

Antibiotics (AB), physical exercise (PE), anxiety (AX), antihypertensive (AH) administration, depression (DL), hormones (HO) administration emerged as relevant elements of the EU constellation of risk factors predicting high/low risk of PTB in clinical context. AB administration can be linked to the presence of an infection during pregnancy [114]. However, the ORACLE trial provided evidence that AB should not be routinely prescribed for women in spontaneous preterm labour without evidence of clinical infection [115]. In our sample, 43 women consumed AB during pregnancy however, 27 of them did not actually have an infection. Our hypothesis is that AB administration during pregnancy likely leads to alterations in the maternal microbiome and previous studies showed that AB treatment to treat maternal infection did not reduce the incidence of PTB [61, 116, 117]. Furthermore, there is increasing evidence that some vaginal microbiomes are associated with an increased risk for PTB [118]and it is feasible that the microbiome alteration caused by AB consumption disrupts the EU environment predisposing for PTB. Moreover, specific components of the feto-placental microbiome have been linked to particular pregnancy complications, some that are strongly associated with PTB: pPROM, FG and PC [114]. PE may instead reduce the risk of PTB by increasing placental perfusion. This decreased risk is presumed to reduce oxidative stress [119], inhibiting the production of maternal and fetal cortisol and reducing the placental production of CRH. This inhibition of CRH prevents onset of uterine contractions and labor. DL has been found to be tightly related to adverse birth outcomes. A meta-analysis of nine studies (with 5,540 women) indicated that the risk of PTB (2.41; 1.47–3.56) was higher in depressed mothers compared to mothers without depression [120]. In general, DL, stress, and AX are associated with an increase in hypothalamic corticotropin-releasing factor (CRF) release and plasma cortisol concentrations. The elevation of placental CRF could likely initiate uterine contractions and cervical ripening [83]. This mechanism in normal, low risk pregnancy may determine a risk increase as it was described. However, AX plays a major role in adherence to treatments and compliance in high-risk pregnancies and may contribute to improve the outcome favoring prevention of noxae of greater magnitude. The relationship between AH administration during pregnancy and PTB risk may be related to possible underlying maternal hypertensive disorders, either before or during pregnancy. Certain AH medication, commonly used during pregnancy, such as beta-blockers, may diminish placental blood flow due to the subsequent vasoconstriction of placental vessels leading to a sort of placental haemodynamic insufficiency consequently leading to potential FG and increasing PTB risk [121]. Since sPTB is likely the final common pathway of several pathogenic processes, a single intervention such as progesterone supplementation is unlikely to benefit all women at risk. This concept was also shown in a major randomized controlled trial for prevention of PTB in women with short cervix in which the beneficial effect of natural progesterone was different according to the degree of cervical shortening [122]. A logistic regression analysis demonstrated significant treatment-genotype interactions, which could result in either a beneficial or harmful treatment response [123]. Studies have also found that women with certain characteristics, such as vaginal bleeding, gonorrhea, or chlamydia in the current pregnancy, a late PTB in a past pregnancy or penultimate PTB, are less likely to have a significant risk reduction [124]. We speculate that, because high-risk PTB women were more likely to deliver preterm, they were also more likely to receive treatment to attempt to delay delivery, such as progesterone supplementation. Finally, medically assisted conception were recently related to an increased risk of PTB both of spontaneous and iatrogenic etiology [22, 125].

### Predictive phase

This study highlighted that a model including only the most informative and relevant selected IU and EU factors is able to classify pregnant women according to the PTB risk dichotomy with a higher accuracy as compared to a model comprising the full list of factors somehow associated to PTB and assessed through the MaFra. A precise constellation of the most informative IU and EU factors significantly impacting on prediction of PTB degree of risk may help guiding clinicians through an otherwise very extensive universe of factors. On one side the IU dimension is a mapped risk territory in the sense that most IU factors are already considered for PTB risk evaluation, on the other hand the EU dimension is populated by many factors related to maternal environment or lifestyle which may only somehow be associated with PTB risk. However, it is extremely important to pinpoint only those EU factors which may considerably add relevant information for the quantification of the degree of risk evaluated only upon clinical groundings in order to increase confidence for attributing PTB risk. In our sample we identified a specific constellation of informative IU and EU factors, however such factor configuration could be on the one hand externally validated on a new sample of mothers or on the other hand alternative configurations may stand out for different populations of pregnant women, therefore we believe that a hierarchical procedure which “explains” the entire gestational dimension on the basis of relevant emerging factors in each dimension for specific populations and able to inform a flexible and dynamic “predictive” model of PTB risk, can guide clinicians through the immense universe of potential factors without losing the focus on PTB risk salient influencers. An increasing number of risk factors are thought to interact to cause a transition from uterine quiescence toward preterm labour and sPTB [29] as well as that from diagnosis to decision of elective iPTB. These risk factors have been found to interact in various ways in the definition of phenotype and etiology of PTB. These factors perhaps aggregate mimicking constellations, thus escalating the potential for a woman to experience PTB. This universe of risk factors, if successfully mapped, could be visualized as a multi-layer model covering the expanse of IU and EU risk factors. This model could provide a more accurate assessment of the pregnancy and evolve into a clinical tool used to calculate the patient-specific risk and their risk specific management or treatment.

### Clustering phase

We have identified three clusters related to degree of PTB risk ranging from high to middle and low, however a probability of group membership is assigned to each pregnant woman in a continuous fashion from 0 to 1, meaning that some women can be almost uniquely assigned to one cluster (i.e. >0.7), however for other women cluster probability membership may be slightly spread among the different clusters. The clustering phase outlined the definition of a frailty profile as a combination of informative IU and EU factors encompassed within the gestational environment (see Fig. 6).

Furthermore, identified clusters mirror the targeting of the delivery time of birth (i.e. gestational week) according to the probability (i.e. membership scores) of falling within the low, medium and high portions of the PTB risk constellation. The high PTB risk group of women clustered around a combination of biological and environmental factors (i.e. IU and EU factors). The middle-risk group clustered around mainly environmental factors (i.e. solely EU factors) without the influence of a pre-existent clinical frailty. It therefore seems that extrauterine factors may have an exclusive weight for the definition of a middle degree of risk and may also have potential impact on childbirth evidenced by the fact that mothers belonging to the middle risk cluster give birth earlier. On the one hand, clusters are clearly characterized by the probability of coexistence of certain risk factors, on the other hand, the effect of the interplay between risk factors more rarely associated should not be underestimated. The specific magnitude of absolute risk for each specific patient will actually be determined by the exploration of these specific associations, which we are willing to assess in future larger studies. For instance, we may claim that short CL added to PP could be informative of a higher degree of risk of PTB as compared to short CL added to maternal AX, so on and so forth. There will be room for future research in exploring these complex constellations of risk factors through Bayesian models of risk predictions or supervised machine learning techniques, the former with an a priori background risk model, the latter with classifiers on a degree of PTB risk continuum. Thus, this study sets the groundings for exploring a more extended universe of risk factors, including for instance the links between abnormal cardiovascular or placental function and PTB and this concept may be followed by the recognition of novel risk factors or potential biomarkers for prediction of PTB. It is known that abnormal cardiovascular adaptation in pregnancy leads to increased risk of preeclampsia, fetal growth restriction and potentially iatrogenic PTB [126]. Recent evidences show that abnormally low uterine arteries pulsatility index is described in pregnancies after in vitro fertilization and frozen blastocyst transfer as well as oocyte donation [22, 125]. In IVF/ICSI group there is on average a higher maternal age and high-risk of both preeclampsia and PTB (spontaneous or iatrogenic). Therefore, novel Doppler observations may help in the definition of specific phenotypes and novel risk factors with potential for generalization to all patients. From a more clinical perspective, frailty profiles may allow to differentiate resources allocation for patients according to their risk composition of IU and EU factors in relation to number of prenatal visits, lab and instrumental investigations such as ultrasound. A subgroup at very low risk would require minimal monitoring and intervention and a very high risk, would require intensive monitoring and treatment. Between these two extremes, at a middle risk PTB risk point, there would be room for continuous patient-specific tailoring of monitoring timing, with a criterion of proportionality according to cluster membership probability values and frailty profiles. For instance, clinicians could appropriately and timely indicate the use of prophylactic betametasone and magnesium sulphate for cases with a specific probability of PTB risk in line with a precise frailty profile, avoiding these treatments for cases below predefined membership values and with a non-overlapping frailty profile. This opens room for the creation of patient-specific risk assessment of PTB and precision medicine approach in this field.

### Clinical translation of the model and future research directions

Obstetrics is going towards early individualization of risks as well as targeted monitoring and interventions [127]. The model described allows longitudinal risk reassessment on repeated visits of individual patients in which the risk background of each new visit would be the posterior risk found in the previous one. Repeated assessment of patients from pre-gestational period to the first trimester may favor preventive strategies for primary prevention. Reassessment at second and third trimester would permit minimizing medicalization in the lower risk groups and increasing surveillance and preparing therapeutic intervention in higher risk groups, proportionally to the risk magnitude of PTB. Thus, we believe that clinical management may be determined by the output of our risk assessment with reproducible and exact quantification. This study opens room to future research on this topic with the aim of accurate patient-specific risk estimation of PTB. This method would also help creating homogeneous study groups on which testing specific prophylactic or therapeutic interventions.

## Conclusions

This study identified with a formal and reproducible methodological framework the selection of relevant and essential IU and EU risk factors able to provide an accurate definition of PTB risk. The first two phase (i.e. explanatory and predictive) identify and cross-validate significant predictors of PTB risk in our sample among both intrauterine factors and extrauterine factors. The third phase (i.e. clustering) spotlights the presence of specific factors which increase (i.e. anxiety) or reduce (i.e. physical exercise) the degree of risk of PTB, suggesting that PTB risk dimensionality is more complex than a binary universe and should be observed through the lens of a preterm birth syndrome in terms of intrauterine-extrauterine interactions that may predispose more or less to preterm birth risk. Founding on the background of IU and EU risk factors, this study establishes a generalized methodology for building-up an evidence-based holistic risk assessment for PTB to be used in clinical practice. Our proposed approach would contribute to increased reproducibility, exact quantification of risk assessment and would help in delivering patient-specific interventions.

## Supplementary Information

**Additional file 1** Detailed overview of the extrauterine (EU) factors selected by Akaike Information Criterion (AIC) in the explanatory phase and the centroids matrix derived from fuzzy C-means clustering on the risk factors selected by AIC in the explanatory phase.

## Data Availability

The dataset generated and analysed during the current study is not publicly available due to ongoing data analysis beyond the scope of this study but is available from cesare.miglioli@unige.ch on reasonable request through a private repository on github. The Maternal Frailty Inventory is available from dellarosa.pasquale@hsr.it on reasonable request.
