# Supplementary material for: A hierarchical procedure to select intrauterine and extrauterine factors for methodological validation of preterm birth risk estimation
Source: BMC Pregnancy Childbirth. 2021 Apr 16;21:306. doi: 10.1186/s12884-021-03654-3 (PMC8052693; doi:10.1186/s12884-021-03654-3)
Supplement: Supplementary file 1 — Additional file 1 Detailed overview of the extrauterine (EU) factors selected by Akaike Information Criterion (AIC) in the explanatory phase and the centroids matrix derived from fuzzy C-means clustering on the risk factors selected by AIC in the explanatory phase. [file 12884_2021_3654_MOESM1_ESM.pdf]

RESEARCH

A hierarchical procedure to select intrauterine and extrauterine factors for methodological validation of preterm birth risk estimation

Pasquale Anthony Della Rosa[1†]
, Cesare Miglioli[2†]
, Martina Caglioni[3]
, Francesca Tiberio[3]
, Kelsey H.H. Mosser[1]
, Edoardo Vignotto[2]
, Matteo Canini[1]
, Cristina Baldoli[1]
, Andrea Falini[1]
, Massimo Candiani[3]
and Paolo Cavoretto[3*]

*Correspondence: cavoretto.paolo@hsr.it
[3]Obstetrics and Gynaecology Department, IRCCS San Raffaele Hospital and University, via Olgettina 62, 20132 Milan, Italy
Full list of author information is available at the end of the article
†Equal contributor

Supplementary Material

In this file we present the additional tables that complete the findings presented in the article and answer to specific comments of the reviewers.

Tables

Table 1 Detailed overview of the extrauterine (EU) factors selected by Akaike Information Criterion [1] in the explanatory phase. We show the proportion of women in each preterm birth risk category depending on the presence of each factor, the estimated beta coefficient of a logistic regression with only the specific EU factor (i.e. univariate $\hat{\beta}$) and the same beta coefficient of a logistic regression done with all the factors (i.e. multivariate $\hat{\beta}$) specified in the EU model. We also provide the standard errors (SE) of the estimates in parenthesis.

EU Factor	Is present?	Low PTB Risk	High PTB Risk	Univariate $\hat{\beta}$ (SE)	Multivariate $\hat{\beta}$ (SE)
EU_AB	No	47 (81%)	11 (19%)	-	-
	Yes	19 (45%)	23 (55%)	1.64 (0.46)	1.07 (0.53)
EU_PE	No	42(57%)	32 (43%)	-	-
	Yes	31 (84%)	6 (16%)	-1.37 (0.50)	-1.45 (0.68)
EU_AX	No	31 (82%)	7 (18%)	-	-
	Yes	41 (57%)	31 (43%)	1.21 (0.48)	0.96 (0.56)
EU_AH	No	63 (70%)	27 (30%)	-	-
	Yes	3 (30%)	7 (70%)	1.69 (0.73)	2.85 (0.98)

Table 2 Matrix of centroids derived from fuzzy C-means clustering [2] on the risk factors selected by Akaike Information Criterion in the explanatory phase. We highlight in bold the risk factors that display at least one discordant sign among clusters (i.e. at least a $+$ and a $-$ on the same column). For continuous variables, the sign represents how much we need to add or subtract to the overall mean to obtain the centroid of a specific cluster. For example, if we consider IU_CL, the typical (i.e. average) woman of Cluster 1 has a cervical length 0.356 cm higher than the overall mean. For dichotomous variables, a positive sign represents a prevalence of subjects that display that characteristic within a given cluster. For example, if we consider EU_AB, the typical woman of Cluster 3 has taken antibiotics since we observe a positive sign. The same reasoning holds for the typical woman of Cluster 2 however, since the coefficient is smaller (i.e. $0.438 > 0.021$), there is a smaller proportion of women that have taken antibiotics with respect to Cluster 3. The risk factors that display at least one discordant sign among clusters are informative.

Cluster	**IU_PP**	IU_PH	**IU_CL**	IU_FG	IU_PC	**EU_AB**	**EU_PE**	**EU_AX**	**EU_AH**
1	-0.018	-0.006	0.356	-0.019	-0.007	-0.223	0.309	-0.207	0.013
2	-0.009	-0.008	0.231	-0.008	-0.015	0.021	-0.177	0.156	-0.025
3	0.018	-0.022	-1.638	-0.070	-0.037	0.438	-0.259	0.137	-0.061

Author details
[1]Neuroradiology Department, IRCCS San Raffaele Hospital and University, via Olgettina 62, 20132 Milan, Italy.
[2]Research Center for Statistics, University of Geneva, Boulevard du Pont-d'Arve 40, 1205 Geneva, Switzerland.
[3]Obstetrics and Gynaecology Department, IRCCS San Raffaele Hospital and University, via Olgettina 62, 20132 Milan, Italy.

References

1. Akaike H. Information theory and an extension of the maximum likelihood principle. In: Selected papers of hirotugu akaike. Springer; 1998. p. 199–213.
2. Bezdek JC, Ehrlich R, Full W. FCM: The fuzzy c-means clustering algorithm. Computers & Geosciences. 1984;10(2-3):191–203.
